# Ethical challenges in connection with the use of coercion: a focus group study of health care personnel in mental health care

**DOI:** 10.1186/1472-6939-15-82

**Published:** 2014-12-04

**Authors:** Marit Helene Hem, Bert Molewijk, Reidar Pedersen

**Affiliations:** Centre for Medical Ethics, Institute of Health and Society, Faculty of Medicine, University of Oslo, P.O. Box 1130, Blindern Oslo, NO-0318 Norway; VU Medical Centre, Department Metamedica, EMGO+, Amsterdam, Netherlands

**Keywords:** Coercion, Ethical challenges, Focus group interview, Mental health care

## Abstract

**Background:**

In recent years, the attention on the use of coercion in mental health care has increased. The use of coercion is common and controversial, and involves many complex ethical challenges. The research question in this study was: What kind of ethical challenges related to the use of coercion do health care practitioners face in their daily clinical work?

**Methods:**

We conducted seven focus group interviews in three mental health care institutions involving 65 multidisciplinary participants from different clinical fields. The interviews were recorded and transcribed verbatim. We analysed the material applying a ‘bricolage’ approach. Basic ethical principles for research ethics were followed. We received permission from the hospitals’ administrations and all health care professionals who participated in the focus group interviews.

**Results:**

Health care practitioners describe ethical dilemmas they face concerning formal, informal and perceived coercion. They provide a complex picture. They have to handle various ethical challenges, not seldom concerning questions of life and death. In every situation, the dignity of the patient is at stake when coercion is considered as morally right, as well as when coercion is not the preferred intervention. The work of the mental health professional is a complicated “moral enterprise”.

The ethical challenges deserve to be identified and handled in a systematic way. This is important for developing the quality of health care, and it is relevant to the current focus on reducing the use of coercion and increasing patient participation. Precise knowledge about ethical challenges is necessary for those who want to develop ethics support in mental health care. Better communication skills among health care professionals and improved therapeutic relationships seem to be vital.

**Conclusions:**

A systematic focus on ethical challenges when dealing with coercion is an important step forward in order to improve health care in the mental health field.

## Background

In recent years, the attention on the use of coercion in mental health care has increased among health care professionals, managers, users/patients, researchers, and politicians. By ‘coercion’, we refer to formal, informal and perceived coercion [1]. In this article, we focus specifically on the many complex ethical challenges which are connected to the use of coercion in mental health care. An ‘ethical challenge’ arises when there is doubt, uncertainty or disagreement about what is right or good [2]. Coercion threatens autonomy of patients, may have adverse effects, and it threatens health care professionals’ perception of what constitutes good care and treatment. Thus, using coercion, while at the same time having the obligation to offer good health care, is a complicated ‘moral enterprise’ which deserves to be systematically examined.

We have identified several studies that explore ethical challenges in an implicit way. Some studies concentrate on difficult feelings and moral distress. For instance, based on in-depth interviews with two psychiatrists, Austin et al. [3] discuss the many competing demands connected to the role of the psychiatrists who were expected to show fidelity both to the public and to patients. The ambiguity connected to their role could easily lead to feelings of moral distress. One study describes bad conscience and fear among health care workers as a result of physically restraining patients [4]. Conflicting intrapersonal feelings concerning the ambiguity created by balancing between acknowledging and correcting the patient is the focus of studies by Vatne et al. [5–7]. Even though there are studies indicating various ethical challenges, the majority of the participants in the study of Lind et al. [8] did not feel that the use of coercion was ethically problematic. The minority, who did find it difficult, felt that forced medication, the use of belts, and isolation were the most problematic (ibid.). In order to cope with using coercion in their work, some employees tend, in different ways, to defend the need to use it [9]. In Bigwood and Crowe’s [4] study, the nurses said that coercion “is part of the job, but it spoils the job”.

In summary, previous research indicates that experiencing ethical challenges in connection with coercion is common among health care practitioners. Our point of departure was therefore that a more explicit and systematic analysis of what kind of moral issues and ethical challenges that arise, could be of great value for health care practitioners in order to improve the quality of care when dealing with coercion.

In this article, we present results from focus group interviews with health care personnel. The interviews focused on the ethical challenges the participants face in their daily practice in mental health care regarding the use of coercion. The research question was: What kind of ethical challenges related to the use of coercion do health care practitioners face in their daily clinical work in mental health care?

## Methods

The present study is part of a larger project about ethical aspects related to the use of coercion in mental health services in Norway (2011–2015). In the study in focus here, we conducted seven focus group interviews with a total of 65 participants (psychiatrists, psychologists, residents, nurses, nursing assistants, social educators, team leaders, and management) from different clinical fields within mental health services (acute wards, rehabilitation units, adolescent psychiatry, geriatric psychiatry, outpatient services), in three different institutions. Those institutions had volunteered to participate in the larger project concerning ethical aspects of the use of coercion. In order for us to gather information about what kind of ethical challenges they face, it was important for us to include employees from all the wards participating in this project. The participants were recruited through the management in the different wards, and we asked for a broad selection of participants regarding age, experience and professional background (purposive sampling). The interviews lasted between 60 and 90 minutes, they were tape recorded and transcribed verbatim (200 pages).

Our interview guide included the following main questions:

- ●What kind of ethical challenges related to the use of coercion do you face in your daily clinical work? For example, have you been in situations where you had doubts about whether coercion should be used or not, or where it felt uncomfortable or wrong? Were there situations where voluntariness was chosen even though there might be equally good reasons for choosing coercion?

Our main aim was to stimulate the participants, with the help of each other, to express freely their experiences, reflections, concerns, and disagreements [10]. In order to avoid general statements, we frequently urged the participants to describe concrete situations in detail.

The reason why we chose to conduct focus group interviews was that we wanted to talk to a large multidisciplinary group of health care professionals, and that we aimed to capture interpersonal dynamics and culture while health care professionals talked about coercion. Focus group interviews are usually conducted by a moderator who will ensure that all voices are heard, the dialogue is based on the subject that is in focus, and that the group’s experiences are expressed through the conversation [11–17]. We chose to have two moderators (first and second author), and we supplemented each other with questions. Moderators must also be alert as to the group dynamics [12, 17]. We were conscious about creating an accepting atmosphere so that the participants would feel free to talk [12]. We followed up with questions for elaboration. To protect patients’ privacy, we asked participants in advance to mask characteristics that may contribute to recognition. During the transcription process, we have also been mindful about changing the names of persons, institutions, and places, as well as considering all information with regard to the risk of identification of individuals [18].

### Data analysis

The analysis is inspired by the concept of ‘bricolage’ [19, 20], which means we have moved freely back and forth in the data material. Our approach was inductive [19]. First, all three authors did a naïve reading of all the transcripts in order to get a first impression of the data material. Each one of us, independent from each other, made a rough outline of what we found interesting and important. This formed the basis of our first discussions. We proceeded by starting to make categories based on our impression of the material. This led to the initial structuring of the material by themes. We made a list of descriptions involving various ethical dilemmas. We have been discussing with each other by sharing our thoughts and impressions, and we have basically agreed to the interpretation of the findings. This means that we have exploited the fact that we are three researchers with both similar and dissimilar theoretical and empirical backgrounds; in total we possess comprehensive knowledge of the health field in general and of the mental health field in particular, clinically and theoretically, as well as regarding research methods. Likewise, we have tested our thoughts and impressions on colleagues in workshops and in presentations on the wards. Importantly, we read an extensive amount of research articles in connection with a literature review on ethical challenges connected to the use of coercion in mental health care which we performed at the same time (work in progress). Through this, we were informed that our study would be an important contribution to this field since there are few studies focusing explicitly on ethical challenges in connection with the use of coercion. Hence, we realised the importance of clarifying what we meant by the concepts ‘ethical challenge’ and ‘coercion’. Through this work we have aimed at meeting both primary (credibility, authenticity, criticality, and integrity) and secondary (explicitness, vividness, creativity, thoroughness, congruence, and sensitivity) criteria of validity [21].

### Ethical considerations

The work was undertaken conforming to the provisions of the Declaration of Helsinki [18], which means that basic ethical principles for research ethics such as informed consent, the right to privacy, respect for personal integrity and dignity [18, 19, 22] were followed. All participants gave informed consent after having received written and oral information about the project. Participant and patient anonymity is preserved in the text. The protocol for the research project has been approved by the Norwegian Social Science Data Services where aspects of privacy protection were assessed [23] (approval September 17, 2012, project number 31360). Since the study does not include patients as participants, we were not, according to Norwegian regulations, obliged to seek approval from the Regional Committee for Medical and Health Research Ethics [24, 25].

## Results

In the presentation of results, we will first describe ethical challenges which seem to occur across various wards and services, related to different types of coercion. Afterwards, we will show how certain challenges are context specific.

### What is coercion and how is it done

Defining coercion is in itself ethically challenging since it has consequences for how the power of health care professionals is recognized and exercised. Defining and recognizing coercion has to do with being morally sensitive and reflective. Several participants reflected on how to understand what coercion actually is. They tended to think of coercion as a broad phenomenon covering many aspects, and they referred to both formal and informal coercion. For instance, one person from an acute ward said:

I have always thought of coercion as involving the big offences, and I have really discovered that coercion is as much a part of the everyday routines, but they are much more difficult to detect.

In order to further reflect upon what coercion is, some contrasted it to the concept of freedom. They stated that we are not entirely free in the way we lead our lives. Being members of society, we have to adjust to regulations and prohibitions, we have obligations at work and in our families and networks, and we react very differently to such “restraints”. Furthermore, some saw the power they have to define what the patients are allowed to do, as ethically challenging. It requires sensitivity and reflectivity which several of our participants talked about.

In interview after interview, they said that it is ethically important how coercion is carried out. It tended to make an essential difference whether coercion was done with a caring and friendly attitude or not, with concern and explanations or not. The way you express yourself, “how you coerce”, as one participant said, is essential to how patients experience the action, whether they feel that their dignity is respected or not. Approaching each patient in an individualised manner seemed to be crucial:

For some … to be admitted here, everything is coercion. To hear keys jingle is the equivalent of the exercise of power. How to tap on the door, right? Do you ask to come in? How to behave? For some, none of what is going on is coercion, while for others everything is coercion.

There is a different and paradoxical kind of ethical challenge that also comes up in the interviews - that challenges common conceptions of coercion - namely that there are patients who request or insist on compulsory admission in the emergency ward, or ask to be put in belts. For example, they may be threatening to commit suicide:

They pull the suicidal card, or they escalate self-harm and do what they need to do in order to be admitted. The ethical dilemma is: should we take care of them on the acute ward or motivate them to take responsibility and offer them help on a lower level (than the acute ward level)?

### Coercion as ‘opportunity’ – formal coercion and conflicting values

For the participants, it seems to be ethically challenging to apply coercion, or their ‘license to coerce’, in good ways. On the one hand, they recognize the opportunities that coercion gives. On the other hand, they are aware of the possibilities for abuse. In two of the interviews (psychogeriatric unit and sub-acute unit), participants said that applying coercion may sometimes be necessary ‘to come into position’ to help a patient. Coercion is the tool that gives the staff the responsibility, possibility, and duty to do good for the patient (beneficence), which is sometimes seen as more important than safeguarding the patient’s autonomy. Another participant (from a psychogeriatric unit) presented the same ethical challenge – the dilemma between paternalism and neglect:

They may say in The Times today that ’old, defenseless woman was removed from her home by force’ or it could be written in The Observer that ’an old lady is perishing in her home and nobody interferes’.

Participants from rehabilitation departments talked about how difficult it can be to support patient autonomy in cases where they have worked intensively for months with patients suffering from both substance abuse and severe mental illness. An example could be when they have been holding back information about the patient having received a considerable amount of money in their bank account (for instance from the tax authorities). The reason for not informing them about this is that they assume that the patient would buy drugs. Consequently, the positive results of months of intensive treatment and care could be destroyed in a very short time. The ethical challenge they face, is how far they can go in utilizing the opportunity to hold back information in connection with coercion – in the name of preventing harm - when this, at the same time, compromises patient autonomy to such a large degree.

The ethical challenges which have been presented so far focus on consequences for individual patients. Yet, another kind of ethical challenge is described by an employee in an acute ward. He is concerned with the relationship between the patient’s right to autonomy, and the protection of both the population and the employees:

It is a dilemma that we are expected to safeguard the patient’s right to autonomy while at the same time the safety of the general public must be respected. We need to think about our employees, they too are entitled to be protected.

The perspective of society is introduced here, and as an ethical challenge the protection of society is quite different from the focus on beneficence and autonomy for individual patients.

### ‘Coercive culture’ in mental health care

By focusing on ethics and coercion in mental health care, we also should be aware of the potentially infringing culture. One participant put it this way:

Even if you do not think about it, there is a tendency in our attitude that ‘I have and you have not, I can leave at 3 pm., you have to stay. I go to the mountains on Friday at 3 pm., ha-ha, you get pizza or porridge tomorrow. We are employees. We wear private clothes, but we also wear id-cards and alarms, we have keys, it is all visible, it is right there, all the time.

The distinction between ‘us and them’ is emphasised by several participants, and some points at the possibility of infringement due to the fundamental asymmetry of power between patients and staff. However, others argued that it is also possible that a culture characterised by asymmetry between patients and staff can uphold dignity by safeguarding the patient’s need to be dependent and receive help. These participants underscored the necessity of being aware of aspects of the culture that can degrade patients and pose a threat to their dignity. As an employee at a mental health district office put it:

It is hard to foster cooperation when the patient only sees you as an abuser.

To exercise care and coercion in a ‘good’ way is challenging since the culture in the mental health field tends to be ‘coercive’.

A different issue mentioned in the focus group interviews is that how coercive routines are actually carried out may vary a lot:

Many of our routines are in themselves limitations to patient autonomy, to be allowed to go outside the ward or not, monitoring, safety procedures, rounds, confiscation of cell phones. The routines and decisions, which are part of everyday life on the ward, are followed up/practiced very differently by the staff.

Another participant (physician) in the same department said:

We find on our rounds between the different wards that coercion is implemented very differently.

Yet, another also talked about

… how coercion is exercised, it could be very different. What kind of vocabulary do you use, how do you relate to the patients, what kind of attitude do you have?

### Informal coercion – relationships and cooperation

Coercion may occur in conflict situations where there might be a weak or non-existing alliance between staff and patient, and where there may be disagreement concerning the participation of the patient in the daily routines:

… we have discussions about this, how long should people be allowed to stay in bed; where, when should we interfere, what do we do?

Informal coercion related to the use of smart phones with internet connection is a topic that comes up several times in the interviews:

It is a real ethical challenge because we set limits for one patient. If we don’t, we risk that pictures taken in the ward can compromise another patient.

Another example of informal, ‘grey-zone’ or ‘fuzzy’ coercion is related to the dosing of forced medication:

… there has been a decision on compulsory drug treatment, and yet you provide such a low dosage that the hospital stay lasts much longer than necessary.

That is, one does not want to give a higher dosage, e.g. to prevent disturbing side-effects. However, this may be more negative because the involuntary hospital admission may last longer.

### Context dependent ethical challenges

As mentioned above, some of the ethical challenges seemed to be dependent upon the context. Such ethical challenges will be presented in the following.

#### Adolescent ward

The adolescent ward faces specific ethical challenges due to the age of their patients and the laws related to age. Parents are supposed to consent on behalf of their children until they are 16 years of age. However, parents do not always know what they agree to:

When the consent from the parents is valid, they are ‘inside’ and begin to influence what is going on. However, to be parents in all this, what are they actually influencing? How many parents have insight into what they are agreeing to when hospitalising their youth? It is not easy. Among other things, in relation to forced tube feeding, it is a major intervention they are involved in and saying ‘yes’ to.

The employees of the adolescent psychiatric department also talk a lot about the difference between the youth being under or over 16 years of age. When the youth turns 16, s/he has reached the legal age, and parents, with some exceptions, are not entitled to insight into the treatment if the patient refuses. This situation is ethically challenging since the health professionals move from including the parents in the treatment the one day to not be allowed to include them the next day. The youth is still the same person with the same needs and challenges. They are still as dependent upon their parents, and usually still live with them. The parents are still supposed to be responsible for their kids, but now without being informed about key aspects of their mental health. It sometimes feels morally wrong to the professionals to exclude the parents.

Employees also described how they exercise coercion or pressure through the parents. An example was when they had asked a father to make sure that his hospitalised son went to bed, something they knew his son refused to do. Other times, parents invade their children and the staff feel they have to protect the patient.

Yet another case they talked about was where the youth had seriously and repeatedly assaulted his parents, something the staff had to stop by force. They also described how they intervene when parents – suffering from guilt towards their kids - smuggle in food and such, and thereby contribute to sabotaging the treatment program. This, among other things, includes the expectation that the patients participate in the daily activities on the ward, like common meals. Several employees also talked about forced tube feeding of adolescents with severe anorexia, which they describe as especially challenging since they have to keep the patient physically fixed.

#### Psychogeriatric ward

Several participants described ethically challenging situations where they manipulate elderly patients through their way of talking to them, for instance in cases where patients say they want to go home. One referred to a patient who said that

… he would like ‘to leave the ship’. So I said, ‘Boy, that’s okay, but first we need to find a place for you to stay and that will probably take some time’. So he agreed to that. ‘Okay’. I mean, we talk to patients that way a lot. The result in this case was that the patient has volunteered to be coercively admitted!

The participants problematised the fact that they are not completely honest in the way they talk to the patients. They express themselves in this way to ensure that their assessment about what is in the best interest of the patient is followed through.

#### Outpatient clinic

A common ethical challenge they face in the outpatient clinic is to observe the patients becoming more and more ill, but refusing to be hospitalised or receive any medical treatment. The health care personnel think that they should wait until the patient is sick enough to be legally committed. The family of the mentally ill person may disagree:

They wanted us to intervene earlier. They wished that they had not had to see how sick their loved one ‘had to become’ before we intervened.

This is a painful ethical challenge for health care professionals. However, they also described how they use the time when coercive measures are not yet taken to build trust and safety in the relationship with the patient, which is important for cooperation in the future, also if coercion is finally needed.

#### Rehabilitation unit

Participants from rehabilitation units presented ethical challenges regarding giving back autonomy to the patient. They described how they spend month after month treating patients – as a rule involuntarily admitted and often medicated against their will – and the patients make huge progress. When the patient’s condition is improving there comes a time when they no longer can be involuntarily admitted. The health care personnel know that the patient might want to quit treatment once the coercive measures are suspended. They also know that the effect of the therapeutic endeavors might be spoiled after a very short time if the patient for instance goes back to drug abuse.

#### Acute ward

A common ethical challenge in the acute wards concerns the urgency and seriousness of the situations. For example, the health professionals described ethical challenges regarding coercion and suicidal patients. One moral question they face is when to let the suicidal patient take back some degree of control of his/her own life? One employee puts the dilemma this way:

So, where is the boundary between what should be the patient’s responsibility in relation to their own lives and what are our responsibilities? Where do these lines cross, for example the extent to which one should dare to give back responsibility to the patient? A while ago, we admitted a young girl who was suicidal. She was in the emergency room for quite some time. She did not want to be admitted. We had discussions with her parents. Her mum was in despair, and finally we decided to let her go. Afterwards, there were a number of suicide attempts and she was brought to the hospital by air ambulance several times. She is ok now, but it is like … these are tough choices. At some point you have to take a chance. But when? What are the consequences? In the worst case, they might die.

As we see here, it is often not obvious what the best option is, and this may lead to disagreement between staff members about whether or not to take over the responsibility, which, in turn, causes inconsistent use of coercion. Sometimes it is the patient’s relatives that are the ones to most strongly oppose giving back some responsibility to the patient.

## Discussion

### Ethical challenges and coercion - a complicated landscape

This study describes the ethical challenges health care practitioners in different parts of the mental health services face. Our findings indicate that coercion in mental health care is a complicated ‘moral enterprise’, encompassing both ‘big dilemmas’ like autonomy versus paternalism, and everyday issues, for example about relationships, communication and cooperation.

### Many important ethical challenges

Since the aim of this study was to explore what mental health professionals themselves understood to be ethical challenges concerning the use of coercion, and since our literature review resulted in few studies on ethical challenges in connection with the use of coercion, we deliberately did not present a fixed definition or theory of ethical challenges. However, in written material and in oral communication with the participants, we said that an ethical challenge occurs when one is unsure of what is the right or good thing to do, and/or when one is not able to do what one thinks is the best thing to do. We considered this understanding to be broad enough to open up for the health care professionals themselves to tell us about the challenges they perceived in their every day clinical work regarding coercion. Our study shows that health care professionals face many important ethical challenges in their daily work with patients and coercion. Not seldom questions of life and death are at stake. In every situation when coercion is considered, the dignity of the patient is at risk. These challenges deserve to be identified and handled in a systematic way within its respective contexts, and with all concerned parties involved. This is important for developing the quality of the health care which is offered, and it is highly relevant to the current focus on reduced and more correct use of coercion. In addition, more precise knowledge about ethical challenges is necessary for those who want to develop ethics support in mental health care.

### Broad understanding of coercion

The study has a broad approach regarding both what is considered to be an ethical challenge and what is coercion. We have included formal, informal, and perceived coercion in the study, which is mirrored in the discussions in the focus groups. It seemed natural for the participants to talk about coercion in the broad sense, and several pointed to subtle forms of coercion, like exercising power or enacting pressure on the patient, what Sjöström [26] names ‘coercion context’ and ‘power dynamics’. We believe that seeing coercion/force/pressure along a continuum helps the staff to develop an awareness of themselves as performing coercion and also power in a broad sense [27], which, in turn, might facilitate willingness to be self-reflective and self-critical.

### Relationships between clinicians and patients in connection with coercion

One important aspect of facing or dealing with ethical challenges in connection with coercion is that it goes on within *relationships* between health care workers and patients: “The clinician-patient relationship and communication may indirectly improve outcome, e.g. mediated through better treatment adherence. Yet, evidence suggests that these interpersonal processes also have a direct therapeutic effect” ([28], p. 521). In a study performed by Theodoridou et al. [29], where they investigated the relationship between perceived coercion and the therapeutic relationship, they found that “perceived coercion predicts the patients’ appraisal of the therapeutic relationship … Perceived coercion is related to a more negative patient-therapist relationship” (p. 939) and perceived loss of autonomy is closely linked to a negative relationship between the clinician and the patient (ibid.). On the other hand, research shows that patients tend to accept the use of coercion if they feel they are treated with respect and are well taken care of [30, 31]. Coercion might involve extra care, attention, safety, and hence contribute to perceived dignity [32, 33]. If patients feel that the staff’s attitude is characterised by beneficence, that the staff is honest and open [34], and they feel that their mental health is improving [30], it is more likely that they will accept the use of coercion. Based on a literature review, van den Hooff [35] finds that being listened to or not being listened to stand out as core experiences determining whether patients feel respected as a human being, or not during coercive admission. Thus, the everyday questions of how to relate in a good way, may sometimes be just as important as determining when deprivation of liberty is legitimate.

### Ethical and professional challenges are closely intertwined

The employees’ awareness of challenges as *ethical* challenges was sometimes not evident. The fact that the challenges they face in their clinical work are presented as ethical challenges, is to some extent a result of us as researchers interpreting them as ethical challenges by applying vocabulary from ethical theory. In the focus group discussions, the challenges often appeared as professional or clinical challenges instead. This said, it is sometimes difficult to strictly differentiate between *professional and moral qualities* because they are closely *intertwined*: “Indeed, the qualities allowing clinicians to be effective in helping patients (clinical skills, knowledge, and attitudes) overlap with, and are, the very qualities that make them morally good. Exemplary clinical practices are in this sense value laden, but also virtue laden” ([36], p. 4). For example, how to use and develop professional competence in a good way, involves both moral and professional issues.

### Lack of an ethics vocabulary and making the implicit explicit

The fact that it seems to be challenging to health care professionals to accurately verbalise ethical challenges might also be understood in the light of lacking theoretical resources and hence lacking normative vocabulary which can illuminate the ethical challenges in a relevant way. Lillemoen and Pedersen [37] point out this fact in their study of ethical challenges in primary health care: “Among the most prominent of this study’s findings is the fact that many of the most frequently experienced ethical challenges are not given much notice in traditional medical and health science ethics and are not even regarded as ethics by many” (p. 104). This statement might encourage us to do studies on clinical practice with the aim to develop and refine a language which is sensitive to – and covers exactly - the ethical challenges in different parts of health care, mental health care included. Also, different kinds of Clinical Ethics Support Services (CESS) might be valuable to assist clinicians in verbalising implicit values inherent in clinical practice, and to go through moral change through dialogue [38]. According to Reiter-Teil et al. [39], it also seems to be important that we address the often implicit notions of ethics which Clinical Ethics Support (CES) rests on. In accordance with Widdershoven et al. [40], who build on hermeneutic ethics, “an approach in which ethics and empirical research inform each other in a cyclical way” (p. 99), is a goal worth striving for.

### Strenghts and limitations of the study

One strength of our study is that we cover various parts of the mental health services, and that different professionals (including psychiatrists and psychologists) and management were included. Both participants with positive and negative or critical attitudes to the use of coercion participated. The fact that we included only employees and not patients is both a limitation and strength: we wanted to learn about how employees perceive ethical challenges, which we did, but we did not learn about the viewpoints of patients. Participant observation, in addition to focus group interviews, could have yielded even more differentiated knowledge about ethical challenges regarding coercion. Our inductive approach yielded rich data on how clinicians struggle with ethical (and professional) challenges in everyday clinical practice. Possibly, a more explicit theoretical framing of the project from the beginning could have led to a clearer conceptualisation of ‘ethical challenges’.

## Conclusion

The use of coercion in mental health care is a complex moral enterprise with frequent and important ethical challenges. These challenges are at least partly dependent on the context, for example the type of services provided. A systematic focus on ethical challenges when dealing with coercion is an important step forward in order to improve health care in the mental health field. We need more research in order to develop knowledge about the kind of activities that might enhance ethical reflection and ethical practice. We believe that a systematic focus on ethics in a broad sense would be fruitful. However, it is sometimes difficult to view ethics and professional questions as separate from each other, in fact, they are in many cases intertwined and should be examined together.

Developing knowledge about ethics in this field is important to better understand the differences *and* connections between ethical and professional issues, so that the focus on and contribution of ethics can be more accurate and precise.
